# Phosphonium-Based Porous Ionic Polymer with Hydroxyl Groups: A Bifunctional and Robust Catalyst for Cycloaddition of CO_2_ into Cyclic Carbonates

**DOI:** 10.3390/polym12030596

**Published:** 2020-03-05

**Authors:** Yizhu Lei, Yali Wan, Wei Zhong, Dingfu Liu, Zhou Yang

**Affiliations:** 1 School of Chemistry and Materials Engineering, Liupanshui Normal University, Liupanshui 553004, China; zw18385142340@163.com (W.Z.); YangZhouand@163.com (Z.Y.); 2 School of Chemistry and Chemical Engineering, Guizhou University, Guiyang 550025, China; liudfgu@163.com

**Keywords:** porous ionic polymer, heterogeneous catalysis, CO_2_ fixation, hydrogen bond donors, phosphonium salt

## Abstract

The integration of synergic hydrogen bond donors and nucleophilic anions that facilitates the ring-opening of epoxide is an effective way to develop an active catalyst for the cycloaddition of CO_2_ with epoxides. In this work, a new heterogeneous catalyst for the cycloaddition of epoxides and CO_2_ into cyclic carbonates based on dual hydroxyls-functionalized polymeric phosphonium bromide (PQPBr-2OH) was presented. Physicochemical characterizations suggested that PQPBr-2OH possessed large surface area, hierarchical pore structure, functional hydroxyl groups, and high density of active sites. Consequently, it behaved as an efficient, recyclable, and metal-free catalyst for the additive and solvent free cycloaddition of epoxides with CO_2_. Comparing the activity of PQPBr-2OH with that of the reference catalysts based on mono and non-hydroxyl functionalized polymeric phosphonium bromides suggested that hydroxyl functionalities in PQPBr-2OH showed a critical promotion effect on its catalytic activity for CO_2_ conversion. Moreover, PQPBr-2OH proved to be quite robust and recyclable. It could be reused at least ten times with only a slight decrease of its initial activity.

## 1. Introduction

With the increasing concerns about environmental crisis, the development of efficient routes for the capture, storage, and utilization of carbon dioxide (CO_2_) that mitigates global warming has attracted tremendous attentions [1,2]. In this context, numerous strategies for mitigating CO_2_ emissions have been proposed, among which the conversion of CO_2_ into useful chemicals appears promising [3,4]. Of various CO_2_ chemical conversions, the catalytic cycloaddition of epoxides and CO_2_ into cyclic carbonates is regarded as one of the most efficient reactions due to its easily available substrates, high selectivity, and 100% atom efficiency [5,6]. At present, many homogeneous catalytic systems have been developed to promote this conversion. Among them, the homogeneous catalysts based on ionic liquids, such as quaternary ammonium salts [7,8], imidazolium salts [9,10,11], and quaternary phosphonium salts [12,13,14], were demonstrated to be the most efficient. Generally, the homogeneous salts could exhibit high catalytic activity under mild reaction conditions; however, the obvious drawbacks of product separation and catalyst recycling still hamper their large-scale applications. Hence, it is desirable to develop highly efficient heterogeneous catalysts for the conversion of epoxides into cyclic carbonates.

Porous organic polymers (POPs), featuring adjustable chemical functionalities and large surface areas, have recently aroused great interest as a class of versatile materials for developing heterogeneous catalysts [15,16]. Nowadays, a number of POPs have been developed for CO_2_ capture and conversion [17,18]. However, the majority of POPs contain monofunctional active sites. Thus, cocatalysts, such as ammonium salts, metal compounds, and/or hydrogen bond donors, are often added for achieving acceptable catalytic activities [18,19]. To heterogenize both catalyst and co-catalyst, bifunctional POPs that consisted of both nucleophiles (halogen anions) and electrophiles (metal salts or hydrogen bond donors) have been recently developed [20,21,22,23,24,25]. Previous reports suggested that POPs that have such dual active sites could cooperatively activate the epoxide, thus improving catalytic performances significantly [20,21,22,23,24,25]. For numerous types of POPs, porous ionic polymers (PiPs) that incorporated ionic moieties into POPs are particularly appropriate for cocatalyst-free CO_2_ fixation [26,27,28,29]. On this account, a number of PiPs with metal and halogen dual active sites have recently emerged as heterogeneous and efficient catalysts for CO_2_ conversion [29,30,31,32]. In contrast, from an environmental viewpoint, the cycloaddition of CO_2_ over a metal-free catalyst is more in line with the requirements of green chemistry because of the low toxicity and easy accessibility of these catalysts [20,26,27,33,34,35,36,37,38]. Therefore, the development of metal-free porous ionic polymer catalysts for the efficient cycloaddition of epoxides and CO_2_ into cyclic carbonates is still highly required.

With this target, a number of imidazolium-based PIPs that contain hydrogen bond donors (HBD) (such as hydroxyl, carboxylic acid, and amine groups) and halogen anions have recently been developed as heterogeneous catalysts for CO_2_ fixation [20,26,27,33,34,35,36,37,38]. Compared with imidazolium-based PIPs, quaternary phosphonium-based PIPs are not as extensively studied due to the less tailored structures and limited heterogenizing method [39,40]. In 2012, Zhang et al. reported that a porous organic polymer containing quaternary phosphonium and tertiary phosphorus (PP-Br) could act as an efficient and recyclable catalyst for the cycloaddition of CO_2_ [41]. In 2015, Wang et al. also reported that in the presence of ZnBr_2_, the phosphonium salt incorporated hypercrosslinked porous polymer displayed high catalytic activity [42]. Later on, Sun et al. synthesized quaternary phosphonium-based PIPs through the free-radical polymerization of vinyl-functionalized quaternary phosphonium salts [43]. The prepared polymeric quaternary phosphonium salts that featured a high density of halide ions exhibited high activities and reusability. Very recently, Xiao et al. reported that polymeric quaternary phosphonium salts containing bromide ion and phenolic hydroxyl group (PPS-*m*OH-Bn) exhibited an excellent synergistic effect for the cycloaddition of epoxides and CO_2_ [44]. The above results indicated that those functionalized phosphonium-based PIPs showed good catalytic performances and reusability for the cycloaddition of CO_2_, especially polymeric quaternary phosphonium salts with hydroxyl groups.

**Scheme 1:**
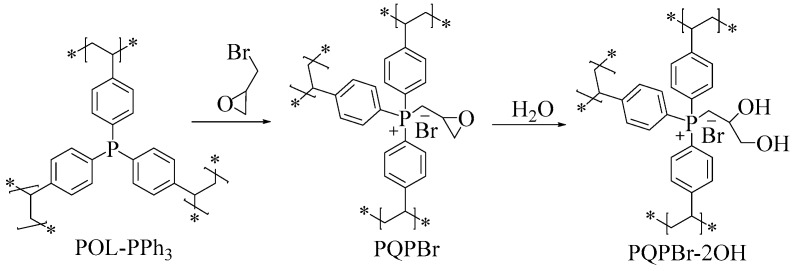
Preparation of the PQPBr-2OH catalyst.

On the other hands, as a kind of easily available and robust HBD group, alkyl hydroxyls have also been widely incorporated in imidazolium-based PIPs [33,34,35,36,37,38] and quaternary phosphonium salts [14,40]; these previous works demonstrated that alkyl hydroxyls showed a nice promoting effect for imidazolium and quaternary phosphonium based catalysts. Inspired by these advances, we envisioned that the fabrication of polymeric quaternary phosphonium salts with alkyl hydroxyl groups might be an efficient way to construct bifunctional and heterogeneous catalysts for the cycloaddition of epoxides and CO_2_. Therefore, we herein designed and fabricated phosphonium-based porous ionic polymer with hydroxyl groups (PQPBr-2OH) as a bifunctional catalyst for CO_2_ fixation. As shown in Scheme 1, PQPBr-2OH was facilely prepared through the reaction of poly(tris(4-vinylphenyl)phosphine) (POL-PPh_3_) and 1-bromo-2,3-epoxypropane, followed by ring opening in hot water. This dual hydroxyls-functionalized polymeric phosphonium bromide has a large surface area and a hierarchical pore structure. Consequently, PQPBr-2OH acted as an efficient, robust, and metal-free solid catalyst for the cycloaddition of epoxides with CO_2_.

## 2. Materials and Methods

### 2.1. Materials

Epoxides, 1-bromo-2,3-epoxypropane, 3-bromo-1-propanol, tetrahydrofuran, and ethanol were purchased from Energy Chemical Company (Shanghai, China). CO_2_ (99.99%) was supplied by a local manufacturer (Liupanshui, China). Tris(4-vinylphenyl)phosphane and poly(tris(4-vinylphenyl)phosphine) (POL-PPh_3_) were prepared as previously reported [45].

### 2.2. Catalyst Preparation

Epoxy-functionalized porous ionic polymer (PQPBr) was prepared via the reaction of poly(tris(4-vinylphenyl)phosphine) (POL-PPh_3_) and 1-bromo-2,3-epoxypropane. Generally, 1.0 g of POL-PPh_3_ was added in 30 mL anhydrous tetrahydrofuran (THF) in a 100 mL pressure-resistant glass tube. After magnetic stirring at room temperature for 2 h, 0.50 g of 1-bromo-2,3-epoxypropane was added to the mixture. Then, the mixture was stirred in an oil bath at 100 °C for 24 h. After the reaction, the white solid was separated by centrifugation, washed with ethanol four times, and dried at 60 °C in a vacuum. The obtained solid was PQPBr. The elemental composition of PQPBr was: C, 68.52%; H, 5.57%; O, 3.58%; P, 6.38%; Br, 14.21%.

Mono hydroxyl-functionalized porous ionic polymer (PQPBr-OH) was prepared via the reaction of poly(tris(4-vinylphenyl)phosphine) (POL-PPh_3_) and 3-bromo-1-propanol. The synthetic procedure of PQPBr-OH was the same as that of PQPBr. The elemental composition of PQPBr-OH was: C, 67.85%; H, 5.95%; O, 3.65%; P, 6.52%; Br, 14.37%.

Dual hydroxyls-functionalized porous ionic polymer (PQPBr-2OH) was prepared by the ring opening reaction in hot water [46]. In a typical procedure, PQPBr (1.0 g) and water (30 mL) were added into a 100 mL pressure-resistant glass tube. The mixture was stirred at 80 °C for 24 h, separated by filtration, and dried at 70 °C in a vacuum oven (YihengDZF-6050, Shanghai Yiheng Technology Co., Ltd., Shanghai, China). The elemental composition of PQPBr-2OH was: C, 65.91%; H, 5.73%; O, 7.02%; P, 6.29%; Br, 14.15%.

### 2.3. Synthesis of Cyclic Carbonates

In a representative experiment, epoxide and PQPBr-2OH (0.05–0.5 mol %, based on Br ion) was introduced into a 15 mL stainless-steel reactor. After purging with CO_2_ three times, the reactor was pressurized with CO_2_ to a low pressure (0.5 MPa). Then, the reaction was proceeded at 60–140 °C for 4 h. After the reaction, the reaction mixture was separated by centrifugation. The resulting solid was washed with ethanol, and dried under vacuum. The liquid mixture was qualitatively and quantitatively analyzed with gas chromatography (GC) and gas chromatography-mass spectrometry (GC-MS).

### 2.4. Catalyst Recycling

The recycling experiment of PQPBr-2OH was carried out in a ten-run cycling experiment. For facilitating the cycling of PQPBr-2OH, the catalytic reaction was performed in scaled-up quantities in a 50 mL stainless-steel reactor. Typically, styrene oxide (12 g, 100 mmol) and the catalyst (0.11 g, 0.2 mol %) were allowed to react at 120 °C for 4 h with a consistent CO_2_ pressure of 0.5 MPa. After each catalytic run, the solid catalyst was recovered by centrifugation, washed with ethanol three times, and dried at 60 °C under vacuum. Then, the recovery PQPBr-2OH was reused in a new catalytic run.

### 2.5. Characterizations

Brunauer-Emmett-Teller (BET) surface areas of the samples were collected on Micrometrics ASAP 2020 (Micromeritics Instrument Co., Norcross, GA, USA) at 77 K. Before the measurements, the samples were vacuum dried at 90 °C for 12 h. Surface morphologies of the obtained samples were observed on scanning electron microscope (SEM, Hitachi S-4800, Hitachi Ltd., Tokyo, Japan) and transmission electron microscopy (TEM, FEI Tecnai G2 F30, Philips-FEI Co., Amsterdam, Netherlands). The contents of CHON elements in the samples were analyzed by an organic elemental analyzer (Elementar Vario MICRO, Elementar Analysensysteme, Hanau, Germany). The contents of P and Br elements were analyzed by inductive coupled plasma optima optical emission spectroscopy (ICP-OES, PerkinElmer, Optima 2000DV, Shelton, WA, USA) and ion chromatograph (Thermofisher ICS-2100, Thermo Fisher Scientific, Sunnyvale, CA, USA), respectively. Before measurement, the polymer was combusted in an oxygen bomb calorimeter and then dissolved in water for analyses. Solid-state ^13^C and ^31^P nuclear magnetic resonance (NMR) spectra were collected on a Bruker AVANCE III 600 Bruker spectrometer (Bruker Corp., Rheinstetten, Germany). X-ray photoelectron spectroscopy (XPS) was conducted on a VG multilab 2000 X-ray photoelectron spectrometer (Thermo Electron Corporation, Waltham, MA, USA). Thermal stability of the sample was tested by thermogravimetric analysis (TGA, NETZSCH STA 449 F5, Netzsch, Serb, Germany) over a temperature range from 30 to 600 °C at a heating rate of 10 °C/min under Ar atmosphere. GC–MS analysis was performed in an Agilent 6890/5973 GC–MS apparatus (Agilent Technologies, Santa Clara, CA, USA). Gas chromatography analysis was carried out by Scientific™ TRACE™ 1310 (Thermo Electron Corporation, Waltham, MA, USA) equipped with an FID detector and a TRACE TR-5MS capillary column.

## 3. Results

### 3.1. Characterizations of the Polymeric Catalysts

**Scheme 2:**
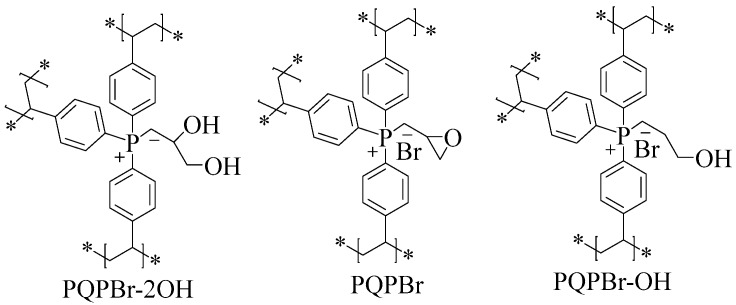
Molecular structure diagrams of the prepared catalysts, * means the long molecular chain.

Dual hydroxyls-functionalized porous ionic polymer (PQPBr-2OH) was prepared by the reaction of poly(tris(4-vinylphenyl)phosphine) (POP-PPh_3_) and 1-bromo-2,3-epoxypropane, followed by the ring opening of the epoxy-tethered precursor (PQPBr) in hot water. For comparison, mono hydroxyl-functionalized porous ionic polymer (PQPBr-OH) was also prepared. The molecular structure diagrams of the three catalysts, PQPBr-2OH, PQPBr, and PQPBr-OH, is illustrated in Scheme 2. The structure, morphologies, and properties of the obtained catalysts were carefully characterized by N_2_ adsorption-desorption, SEM, TEM, FT-IR, elemental analysis, Solid-state ^13^C NMR and ^31^P NMR spectra, XPS, SEM-mapping, and TGA.

The porous properties of PQPBr, PQPBr-OH, and PQPBr-2OH were analyzed by nitrogen sorption analysis. As shown in Table 1, POP-PPh_3_ showed a high BET surface area of 1146 m^2^ g^−1^ and a pore volume of 2.41 cm^3^ g^−1^. After functionalization with epoxy, mono hydroxyl, and dual hydroxyls groups, the BET surface areas and pore volumes of the resulting PQPBr, PQPBr-OH, and PQPBr-2OH obviously decreased. These decreases of physical properties could be assigned to the partial pore filling after functionalization with alkylation groups. In spite of the obvious decrease in BET surface areas with this post-modification method, the obtained PQPBr, PQPBr-OH, and PQPBr-2OH still possessed higher surface areas than that of polymeric quaternary phosphonium salts preparing by direct polymerization of vinyl-functionalized quaternary phosphonium salts in *N,N*-dimethylformamide (DMF) solvent [43,44]. These high BET surface areas might be attributed to the use of THF as a solvent for the polymerization and alkylation reactions; THF showed excellent swelling properties towards the phosphine-functionalized porous polymer that was prepared by the polymerization of vinyl-functionalized monomers [47,48]. 

**Figure 1:**
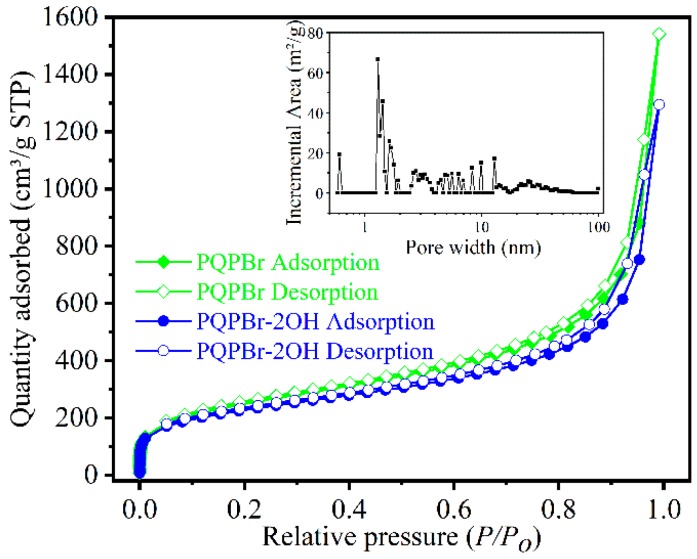
Nitrogen sorption isotherms of PQPBr and PQPBr-2OH, and pore size distribution curve (**inset**) of PQPBr-2OH.

Nitrogen sorption isotherms of PQPBr and PQPBr-2OH are shown in Figure 1. They showed the combined type I and type IV isotherms. The steep steps of absorption curves at low relative pressure (*P/P*_0_ < 0.01) indicated the abundance of micropores. The sharp rising adsorptions at high relative pressure regions (0.7 < *P/P*_0_ < 1.0) suggested the existence of macropores, while the presence of the hysteresis loops at *P/P*_0_ = 0.8–1.0 regions reflected a certain amount of mesopores. Pore size distribution curve of the representative PQPBr-2OH in Figure 1 (inset) further demonstrated its hierarchically porous structure that was comprised of a large amount of micropores and mesopores, and a small quantity of macropores. For heterogeneous catalysts, the big surface area and high pore volume of the material could maximize the accessibility of the catalytically active sites toward the substrates, while the hierarchically porous structure of the material could enhance the mass transfer of the reactants and products. Therefore, it is not unreasonable to expect that the prepared PQPBr-2OH could be a promising choice of heterogeneous catalyst due to its big surface area, large pore volume, and hierarchical pore structure.

**Figure 2:**
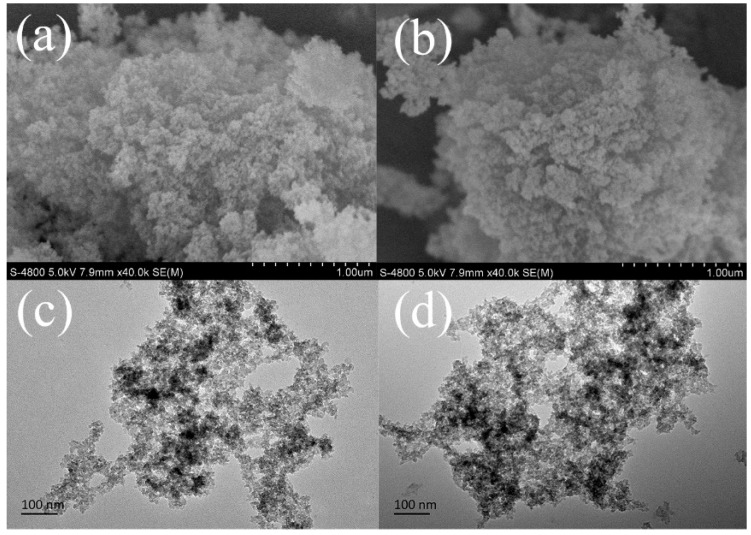
Scanning electron microscope (SEM) images of (**a**) PQPBr and (**b**) PQPBr-2OH; Transmission electron microscopy (TEM) image of (**c**) PQPBr and (**d**) PQPBr-2OH.

SEM and TEM were used to study the surface morphologies of PQPBr and PQPBr-2OH. As shown in Figure 2, the representative SEM images of PQPBr and PQPBr-2OH illustrated that the two samples had a similar sponge-like morphology that was composed of small and loosely aggregated particles. TEM images in Figure 2c,d further confirmed the porous and loosely packed morphologies of PQPBr and PQPBr-2OH.

**Figure 3:**
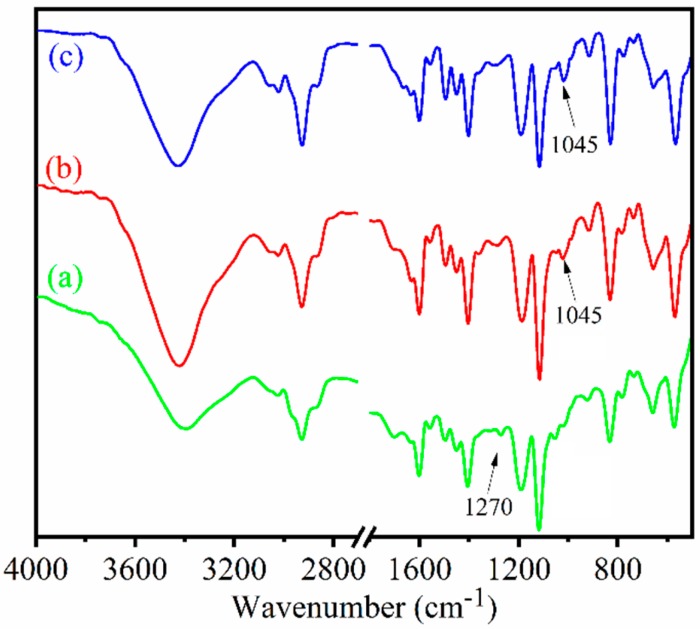
Fourier transformed infrared (FT-IR) spectra of (**a**) PQPBr, (**b**) PQPBr-OH, and (**c**) PQPBr-2OH.

Figure 3 shows the FT-IR spectra of PQPBr, PQPBr-OH, and PQPBr-2OH. The three samples displayed a series of bands at around 2950–2820 and 1600–1450 cm^−1^, which corresponded to C–H stretching vibrations of the saturated alkyl chains and skeleton stretching of benzene rings, respectively. The FT-IR spectrum of PQPBr in Figure 3a shows a characteristic peak at 1270 cm^−1^, which could be assigned to the C−O stretching vibration of the epoxy groups [49]. After the ring opening reaction in hot water, the characteristic peak at 1270 cm^−1^ disappeared, and a new characteristic peak of OH groups at around 1045 cm^−1^ [50] was present in the FT-IR spectrum of PQPBr-2OH.

Elemental analysis was used to investigate the elemental contents of the samples. As shown in Table 2, PQPBr showed P and Br contents of 2.06 mmol/g and 1.78 mmol/g, respectively. As calculated from the mole ratio of Br/P, about 87% of phosphine was alkylated with 1-bromo-2,3-epoxypropane in PQPBr. PQPBr showed an O content of 2.2 mmol/g. After the ring opening reaction in hot water, the resulting PQPBr-2OH showed an O content of 4.3 mmol/g, which was about two times of that in PQPBr. This result indicated that the epoxy groups were almost totally hydrolyzed in PQPBr-2OH. Elemental analysis of PQPBr-OH also suggested that the majority of phosphine was alkylated with 1-bromo-2,3-epoxypropane. Elemental analyses of the three samples showed that the experimental values of O were slightly higher than the corresponding theoretical values; this deviation might be mainly attributed to the presence of trapped guest molecules (e.g., water), which is common in porous materials.

**Figure 4:**
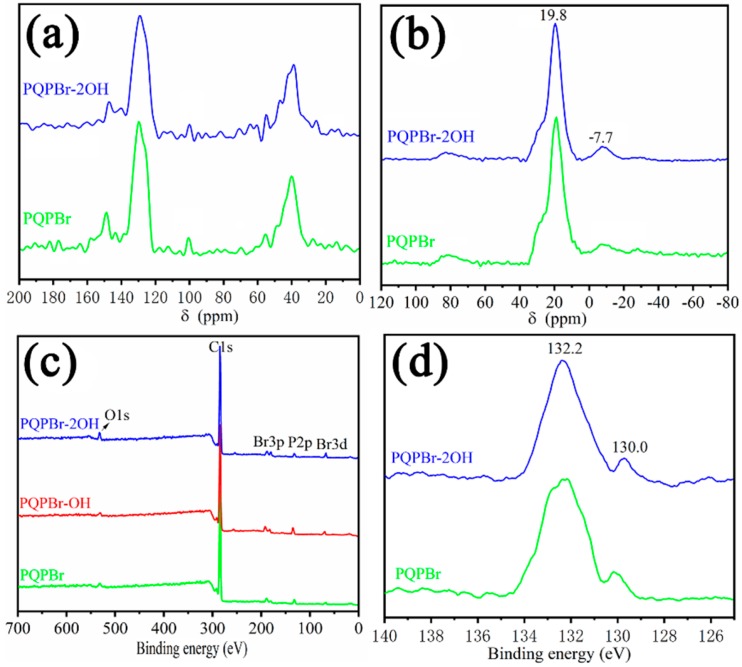
(**a**) Solid-state ^13^C nuclear magnetic resonance (NMR) spectra of PQPBr and PQPBr-2OH; (**b**) solid-state ^31^P NMR spectra of PQPBr and PQPBr-2OH; (**c**) XPS full spectra of PQPBr, PQPBr-OH and PQPBr-2OH; (**d**) P 2p XPS spectra of PQPBr and PQPBr-2OH.

**Figure 5:**
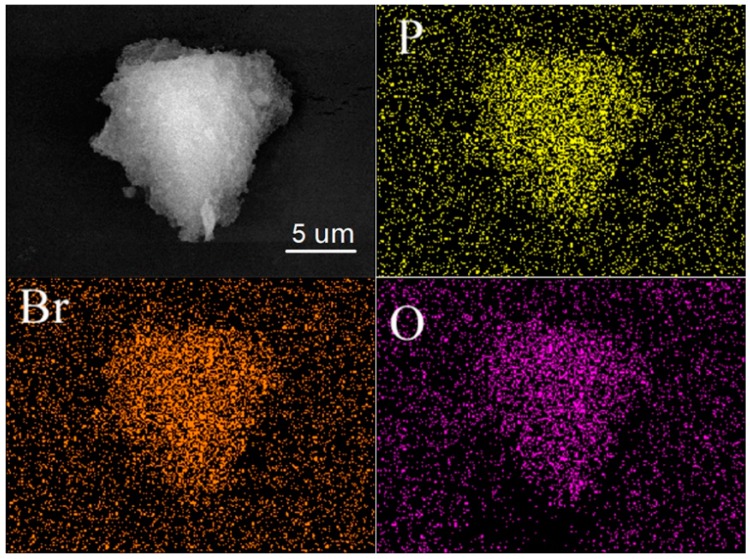
SEM-mapping of PQPBr-2OH.

To gain insight into the structural information of the prepared samples, solid-state ^13^C NMR, solid-state ^31^P NMR, and XPS analyses were carried out. The solid-state ^13^C NMR spectra of PQPBr and PQPBr-2OH in Figure 4a displayed a number of peaks at around 120–150 and 30–70 ppm, corresponding respectively to the carbons of benzene rings and saturated carbon chains. Solid-state ^31^P NMR spectra of PQPBr and PQPBr-2OH in Figure 4b showed a main peak with a small peak at around 19.8 ppm and −7.7 ppm, respectively. The peak at 19.8 ppm can be attributed to the phosphorus atoms of the phosphonium salts [45,51], while the small peak at around −7.7 ppm should be assigned to a small amount of unfunctionalized phosphine [44,51]. These findings further indicated that most of P atoms in PQPBr and PQPBr-2OH was successfully alkylated by alkyl bromide. The XPS full spectra of PQPBr, PQPBr-OH, and PQPBr-2OH in Figure 4c confirmed the presence of O, C, Br, and P elements in the three samples. Compared to PQPBr, PQPBr-2OH exhibited a higher relative intensity of the O1s band, thus verifying its relatively higher O content. P2p XPS spectra of PQPBr and PQPBr-2OH in Figure 4d displayed a characteristic band of phosphonium salt at 132.2 eV. The small band (130.0 eV) near the main energy band gap might be assigned to a small amount of unfunctionalized PPh_3_ units. Thus, solid-state ^31^P NMR and XPS analyses further demonstrated the successful fabrication of dual hydroxyls-functionalized porous ionic polymers (PQPBr-2OH). Scanning electron microscopy (SEM) elemental mapping was used to investigate the elemental dispersion of the PQPBr-2OH sample. As shown in Figure 5, P, Br, and O elements were well distributed in the PQPBr-2OH polymer with high degrees of dispersion.

**Figure 6:**
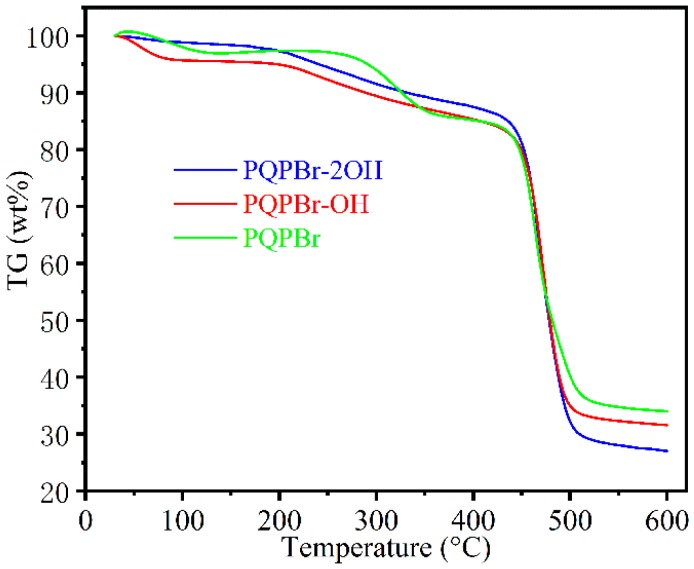
Thermogravimetric analysis (TGA) curves of PQPBr, PQPBr-OH, and PQPBr-2OH.

Thermal stability of the sample was examined by thermogravimetric analysis (TGA) over a temperature range of 30 to 600 °C at a heating rate of 10 °C/min under Ar atmosphere. As depicted in Figure 6, PQPBr-OH and PQPBr-2OH showed similar TGA curves. The initial weight loss that occurred at 25–150 °C should be mainly ascribed to the removal of guest molecules. The rapid weight losses took place at above 200 °C, suggesting that the two samples could be stable at least up to a temperature no less than 200 °C.

### 3.2. Catalytic Activity

With the expected materials in hand, their catalytic performances for the cycloaddition of epoxides with CO_2_ were investigated. Firstly, the catalytic activities of different catalysts were first investigated using the cycloaddition of inactive styrene oxide as a model reaction. As shown in Table 3, under the mild reaction conditions of 0.5 MPa CO_2_ pressure, 100 °C, and 0.5 mol % catalyst loading, PQPBr, PQPBr-OH, and PQPBr-2OH could promote the cycloaddition of inactive styrene oxide in 41%, 75%, and 89% yields, respectively (Table 3, entries 1–3). It was obvious that PQPBr-2OH showed higher activity than that of PQPBr and PQPBr-OH catalysts. In terms of their similar surface areas and ionic concentrations, the differences in catalytic activities should be attributed to the promoting effect of HBD groups (hydroxyl groups). PQPBr-2OH, having both Br anions and hydroxyl groups, could cooperatively activate the epoxide, thus improving catalytic performances significantly [20,26,27,33,34,35,36,37,38]. Moreover, the concentrations of the hydroxyl groups also affected the catalytic performance, and the PQPBr-2OH catalyst with dual hydroxyls exhibited higher catalytic activity than PQPBr-OH with one hydroxyl, suggesting that the OH groups activated the epoxide more easily through multiple hydrogen bonding and, thus, enhanced catalytic efficiencies [8,52]. Under the same conditions, the nonionic polymer POP-PPh_3_ gave a negligible yield of cyclic carbonate, verifying that catalytic active sites originated from the ionic moiety (Table 3, entry 4). Previous reports indicated that the reaction temperature had an important effect on the catalytic performance of the catalysts for the cycloaddition of epoxides with CO_2_ [33,34,35,36,37,38]. Therefore, the influence of reaction temperature was studied (Table 3, entries 5–8). With the reaction temperature rising from 60 to 120 °C, the yields of target cyclic carbonate were significantly increased, and an almost quantitative yield was obtained at 120 °C. Further increasing the reaction temperature to 140 °C, a slight decrease in selectivity was observed with a minute amount of 1,2-propanediol generated as a by-product. At 120 °C, a lower catalyst loading was also tested. Interestingly, a 98% yield of cyclic carbonate and a TOF number of 123 h^−1^ could be obtained using a low catalyst loading of 0.2 mol % (Table 3, entry 9).

Next, the scope of the PQPBr-2OH catalyst for the cycloaddition of epoxides and CO_2_ was investigated with several typical epoxides. As shown in Table 4, with a 0.05 mol % catalyst loading, PQPBr-2OH could effectively convert these typical epoxides into the corresponding cyclic carbonates in moderate to high yields. Moreover, despite the relatively high steric hindrance and low-reactivity, cyclohexene oxide could also be smoothly converted at low catalyst loading (Table 4, entries 8 and 9).

**Figure 7:**
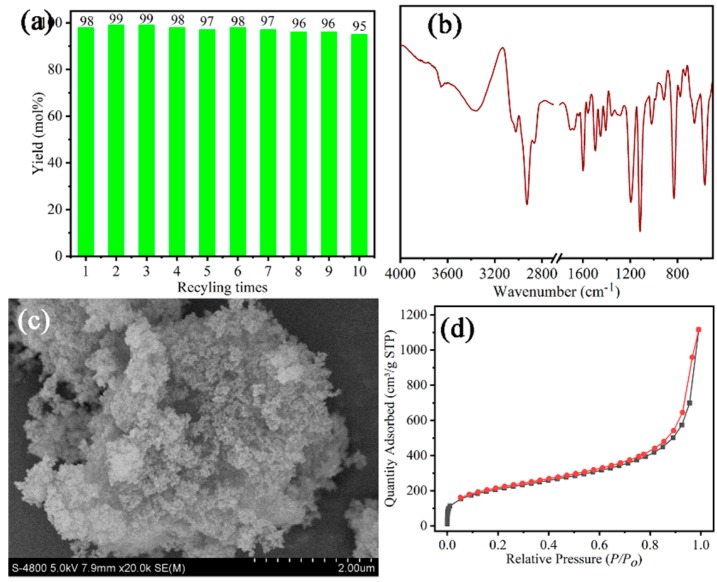
(**a**) Catalytic reusability of PQPBr-2OH. Characterizations of the recycling of the PQPBr-2OH catalyst after reusing it ten times: (**b**) FT-IR, (**c**) SEM, and (**d**) Nitrogen sorption isotherms.

Then, stability and recyclability of PQPBr-2OH were tested in a ten-run recycling experiment. For facilitating the recycling of PQPBr-2OH, catalytic reactions were performed in scaled-up quantities using styrene oxide (12 g, 100 mmol) and PQPBr-2OH (0.11 g, 0.2 mol %). Noticeably, even with ten times the amount of styrene oxide, a 99% yield of target cyclic carbonate could be gained under the reaction conditions. After the first reaction, the solid catalyst was recovered by centrifugation, washed with ethanol, dried under vacuum, and then directly reused in a new catalytic run. The results in Figure 7a revealed that PQPBr-2OH exhibited excellent reusability for the cycloaddition of styrene oxide and CO_2_. After reusing it 10 times, a 95% yield of target cyclic carbonate was still preserved. Moreover, the chemical structure and morphology of the recycling PQPBr-2OH after reusing ten times were analyzed by FT-IR, SEM, and N_2_ adsorption-desorption (Figure 7b–d). The results suggested that the chemical structure and morphology of the recycling PQPBr-2OH were well maintained, thus demonstrating the robustness of the prepared catalyst.

### 3.3. Plausible Reaction Mechanism

**Figure 8:**
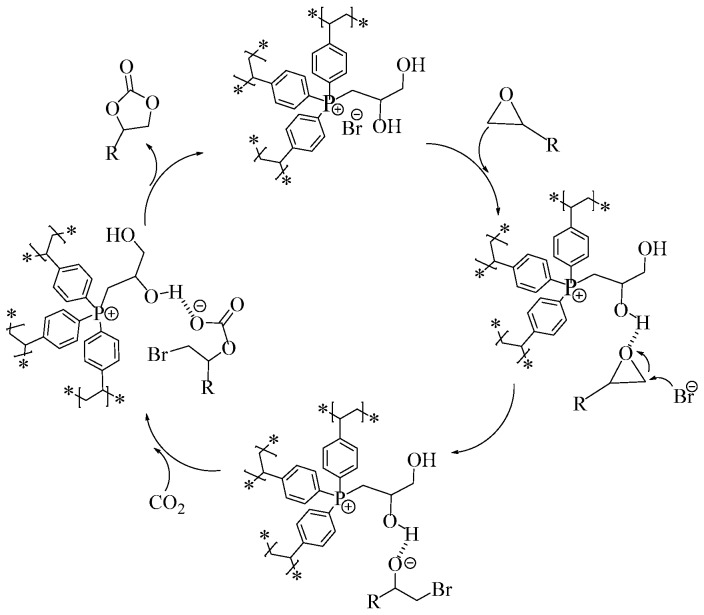
Proposed reaction mechanism for the cycloaddition of CO_2_ to epoxides catalyzed by PQPBr-2OH.

According to the above results, PQPBr-2OH with hydroxyl groups exhibited higher catalytic activity than that with non-hydroxyl groups. These findings indicated that hydroxyl groups have an important promoting effect for the cycloaddition of CO_2_ to epoxide. Moreover, the nucleophilic attack of bromide anion on epoxide is essential. Previous reports suggested that the ring-opening step of epoxide is rate-determining [34,53,54], and a nucleophilic attack of an anion on the carbon atom of epoxide with a synergetic electrophilic attack of a cation on the oxygen atom of epoxide can facilitate this step significantly [20,26,27,33,34,35,36,37,38]. Moreover, it was also well demonstrated that the hydrogen bond between the hydroxyl group and epoxide has a good promoting effect for the ring-opening step, thus accelerating this cycloaddition reaction [34,35,36,37,38,40,46,52,53]. On the basis of the experimental results and these pioneering reports, a plausible mechanism was proposed and shown in Figure 8. First, hydrogen bonding was formed between the hydroxyl (any one of the dual hydroxyls) of QPBr-2OH and the oxygen atom of epoxide. Then, the bromide anion of PQPBr-2OH attacked the less sterically hindered carbon atom of the epoxide, thus opening the epoxy ring to form a haloalkoxy intermediate. Thereafter, haloalkoxy intermediate reacted with CO_2_ to produce an alkyl carbonate, which subsequently transformed to cyclic carbonate via a ring-closure step.

## 4. Conclusions

In summary, a phosphonium-based porous ionic polymer (PQPBr-2OH) with a large surface area, hierarchical pore structure, functional hydroxyl groups, and high density of active sites was developed as an efficient and robust catalyst for the cycloaddition of epoxides with CO_2_. Control experiments based on the reference catalysts revealed the enhancement achieved by the introduction of dual hydroxyl functionalities in PQPBr-2OH catalyst. Thus, with a low catalyst loading, high yields of cyclic carbonates were obtained under a low CO_2_ pressure. In addition, PQPBr-2OH was quite robust and recyclable and could be reused at least ten times without obvious change of chemical structure and morphology. Thus, this study provided not only a promising catalyst for the cycloaddition of epoxides with CO_2_, but also some clues to develop bifunctional and robust phosphonium-based PIPs for CO_2_ fixation.

## Figures and Tables

**Table 1:** Textural properties and Br^–^ contents of the prepared catalysts.

Entry	Sample	*S*_BET_ (m^2^ g^−1^) *^a^*	*V*_p_ (cm^3^ g^−1^) *^b^*	D_ave_ (nm) *^c^*
1	POP-PPh_3_	1146	2.41	8.42
2	PQPBr	879	2.20	6.25
3	PQPBr-OH	848	1.57	3.39
4	PQPBr-2OH	794	1.90	6.26

*^a^* BET surface area. *^b^* Single point adsorption total pore volume of pores at *P/Po* = 0.95. *^c^* Adsorption average pore diameter (4V/A by BET).

**Table 2:** The elemental contents of the prepared samples.^a^

Sample	P (mmol/g)	Br (mmol/g)	O (mmol/g)
PQPBr	2.06	1.78	2.2
PQPBr-OH	2.10	1.80	2.3
PQPBr-2OH	2.03	1.77	4.3

^a^ Average experimental value of three parallel experiments.

**Table 3:**
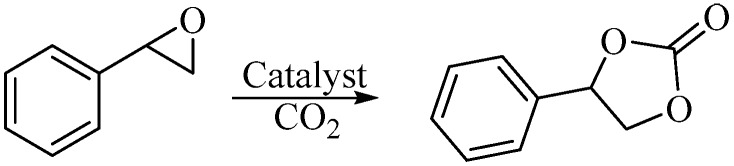
Effects of catalysts and reaction temperature.^a^

Entry	Sample	T (°C)	Yield (mol %)	Selectivity (mol %)	TOF (h^−1^)
1	PQPBr	100	41	>99	20.5
2	PQPBr-OH	100	75	>99	37.5
3	PQPBr-2OH	100	89	>99	44.5
4	POP-PPh_3_	100	<3	-	-
5	PQPBr-2OH	60	7	>99	3.5
6	PQPBr-2OH	80	36	>99	18
7	PQPBr-2OH	120	99	>99	49.5
8	PQPBr-2OH	140	98	98	49
9 ^b^	PQPBr-2OH	120	98	>99	123

^a^ Reaction conditions: styrene oxide (1.2 g, 10 mmol), catalyst (0.5 mol %), CO_2_ (0.5 MPa), 4 h, 800 rpm. ^b^ PQPBr-2OH (0.2 mol %).

**Table 4:** Cycloaddition of CO_2_ with various epoxides using PQPBr-2OH as a catalyst.^a.^

Entry	Substrate	Product	Catalyst Loading (mol %)	Yield (mol %)	Selectivity (mol %)
1	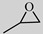	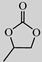	0.2	99	>99
2			0.1	99	>99
3			0.05	98	>99
4	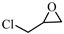	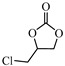	0.1	99	>99
5			0.05	91	>99
6	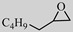	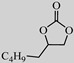	0.1	99	>99
7			0.05	95	>99
8	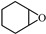	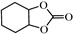	0.1	99	>99
9			0.05	74	>99

^a^ Reaction conditions: epoxide (10 mmol), PQPBr-2OH, CO_2_ (0.5 MPa), 120 °C, 4 h, 800 rpm.
